# Public Health and School Sanitation

**Published:** 1896-06

**Authors:** Ida C. Bender, Jane North Frear

**Affiliations:** Buffalo; Buffalo


### PUBLIC HEALTH AND SCHOOL SANITATION.

By IDA C. BENDER, M. D., Buffalo, assisted by JANE NORTH FREAR, M. D., Buffalo.

### HEALTHFULNESS OF BUFFALO.

UNDER this heading the *Journal of the Amer. Med. Assn.*, May 9th, makes some interesting comments regarding the sources of error in computing the death-rate of cities on the basis of an estimated population, citing the following instances : "In New York, for example, up to April 27, 1895, the population was 'estimated' at 2,013,723, and the death-rate for the week ending on that date—the total number of deaths reported being 885—was figured at 22.93 per annum in the weekly report of the New York Health Department. In the report of the following week this rate shot up to 25.7, while the deaths reported were 890, or only *five* more than for the previous week. The explanation is found in a foot-note on the first page of the May 4th report : 'Police census, April 15, 1895, 1,849,866'—a shrinkage of more than 7 per cent. in the 'estimated' population." Similar inaccuracies are shown to have occurred in Chicago for the same reason. The *Journal* then goes on to note with favor the method in use in the Buffalo Department of Health, which consists not only of the publication of a rate based upon a population, estimated with more or less care, but also of a statement of the actual number of deaths reported year by year. The latter figures show a substantial reduction in the past five years, namely : 1891, deaths 6,001, rate 23.48 ; 1892, 5,697, 19.98 ; 1893, 5,711, 19.03 ; 1894, 5,280, 16.76 ; 1895, 4,684, 13.95.

---

### A HEALTH DEPARTMENT OF THE RENAISSANCE.

IN *Annales de la Societé Méd.-Chir. de Liége*, February, 1896, we read an account of a Bureau of Health in the city of Lyons, France, as early as 300 years ago, which was much more than a prophecy of the modern but hardly less advanced and successful department of to-day. Among its regulations were those providing for the declaration of contagious diseases and the disinfection of residence, clothing and bedding. It is a noteworthy fact that the disinfecting materials employed included some of our best modern antiseptics—sublimate, arsenic, camphor and various essences.

---

### THE COST OF AN EPIDEMIC.

In the *Popular Science Monthly* for March the following interesting statistics, compiled by Dr. Munro, are given. They were first published in the *British Medical Journal.* In the course of an epidemic of enteric fever, in 1893, there occurred 859 cases and 74 of these died. The loss in wages was $16,455 ; the cost of treatment, $21,475 and of funeral expenses $1,850.

Adopting Farr's estimate of the average value of each wage-earner as $795, we have for 74 deaths the sum of $58,830. By this estimate the pecuniary loss to the community arising in connection with the epidemic was $98,610.

Dr. Munro says that a consideration of these figures may well suggest the question whether any investment is calculated to yield a better pecuniary return than money spent in liberally maintaining a well-equipped public health department, which has for one of its chief objects the prevention of epidemics.

---

### MEDICAL INSPECTION IN BOSTON SCHOOLS.

Durgin (*Boston Medical and Surgical Journal*, April 9, 1896,) gives an account of the work recently taken up by the Board of Health of Boston, to secure medical inspection of schools and supervision of the isolation and release of persons suffering from diphtheria and scarlet fever. The board of health divided the city into fifty districts, with an average of four school-houses and 1,400 pupils to each district. It appointed one physician for each district, whose duty it is to visit the schools in his charge daily, soon after the beginning of the morning session. Teachers are instructed to note the appearance of symptoms of illness among the pupils and to report the same to the visiting physician. The latter examines the reported children, making a record of his diagnosis and action in books furnished by the health board and kept in the custody of the master of each school. Sick children are sent home to be cared for by parents and the family physician. If it is found that a child has a contagious disease, such child is ordered home and the case reported to the health board. A wooden tongue depressor is used for examination of the throat. Each depressor is used once and then burned. The medical inspector never undertakes to give professional treatment in any case; he merely points out the need of treatment and this must be received from the family physician or in hospitals or dispensaries. The total number of children examined between November 1, 1894, and October 31, 1895, was 14,666, of whom 9,188 were found to be sick. The number found sick enough to be sent home was 1,745. Of these, 437 were suffering from contagious or infectious diseases, as follows: diphtheria, 70; scarlet fever, 26; measles, 110; whooping-cough, 28; mumps, 43; pediculosis, 66; scabies, 42; congenital syphilis, 8; chicken-pox, 34. Many other diseases were discovered needing treatment, such as swollen glands, anemia, chorea, Potts's disease, and the like.

Incidental to this school inspection the same men also serve as agents of the board of health in the control of contagious diseases treated at home. Each medical inspector is held responsible for the proper isolation of the patient at home, for causing the patient's removal to the hospital when necessary and for the patient's release from isolation. The board of health is thus provided with trustworthy information, upon which it can act for the protection of, the schools and the public against the spread of contagious diseases.

---

Queen Margaret College, Glasgow, the women's department of the university, has enrolled 67 medical students. Of the graduates of medicine and surgery of this college 2 are in practice in Glasgow, 5 are engaged in foreign mission work in India, 1 is in Egypt and 2 are ready to proceed to China.—*Brit. Med. Jour.*—*Med. Review.*

---

A school of medicine for women, at St. Petersburg, has already an annual grant of $50,000 from the government and $12,000 from the city, while from private sources the sum of $360,000 has been raised.—*Med. Record.*

---

A woman occupies the chair of histology in the medical faculty of Bologna.

